# Ligation-anchored PCR unveils immune repertoire of TCR-beta from whole blood

**DOI:** 10.1186/s12896-015-0153-9

**Published:** 2015-05-28

**Authors:** Fan Gao, Kai Wang

**Affiliations:** Zilkha Neurogenetic Institute, University of Southern California, 1501 San Pablo Street, Los Angeles, CA 90089 USA; Department of Psychiatry, University of Southern California, Los Angeles, CA 90089 USA; Current address: The Picower Institute for Learning and Memory, Massachusetts Institute of Technology, Boston, MA 02139 USA

**Keywords:** Next-generation sequencing, Immune repertoire, Blood

## Abstract

**Background:**

As one of the genetic mechanisms for adaptive immunity, V(D)J recombination generates an enormous repertoire of T-cell receptors (TCRs). With the development of high-throughput sequencing techniques, systematic exploration of V(D)J recombination becomes possible. Multiplex PCR has been previously developed to assay immune repertoire; however, the use of primer pools leads to inherent biases in target amplification. In our study, we developed a “single-primer" ligation-anchored PCR method that may amplify the repertoire with much less biases.

**Results:**

By utilizing a universal primer paired with a single primer targeting the conserved constant region, we amplified TCR-beta (TRB) variable regions from total RNA extracted from blood. Next-generation sequencing libraries were then prepared for Illumina HiSeq 2500 sequencer, which generates 151-bp read length to cover the entire V(D)J recombination region. We evaluated this approach on blood samples from healthy donors and from patients with malignant and benign meningiomas. Mapping of sequencing data showed that 64% to 96% of mapped TCRV-containing reads belong to TRB subtype. An increased usage of specific V segments and V-J pairing were observed in malignant meningiomas samples. The CDR3 sequences of the expanded V-J pairs were distinct in each malignant individual, even for pairing of TRBV7-3 with TRBJ2-2 that showed increased usage in both cases.

**Conclusions:**

We demonstrated the technical feasibility and effectiveness of ligation-anchored PCR approach in capturing the TCR-beta landscapes. Further development of this technology may enable a comprehensive delineation of immune repertoire, including other forms of TCRs as well as immunoglobulins.

**Electronic supplementary material:**

The online version of this article (doi:10.1186/s12896-015-0153-9) contains supplementary material, which is available to authorized users.

## Background

Activation of the immune system, together with genomic alternation, including somatic hypermutation and recombination, establishes innate and adaptive immunity in the immune system [1,2]. Both the immunoglobulin (Ig) and the T cell receptor (TCR) loci contain many different V, D and J segments, which are subject to a tightly regulated genomic rearrangement process – V(D)J recombination – during early lymphoid differentiation [3-5]. For a given TCR subtype, complementarity determining region 3 (CDR3) is generated by the V(D)J combination at this subtype locus, with the translated protein sequence forming the center of the antigen binding site [6], defining the affinity and specificity of the receptor for individual peptide-MHC complexes. The CDR3 sequence of dominant clones in cancer patient may serve as a signature to diagnose cancer or to classify tumor into subtypes. In addition, signatures could be obtained at the time of disease diagnosis and then monitored on an ongoing basis, to assess the effects of anticancer therapies or for early detection of recurrence [7,8].

The importance of monitoring TCR in human health and disease has been increasingly recognized. Recent studies showed that TCR repertoire has been found to affect a wide range of diseases, including malignancy, autoimmune disorders and infectious diseases, and, given the broad involvement of the immune system in almost all of human health and disease, this reach should be expected to expand greatly [9]. However, conventional methods to measure V(D)J recombination have several limitations to prevent detailed characterization of immune repertoire. More recent approaches, such as multiparameter flow cytometry, spectrotyping, or custom-designed real-time PCR assays, are slightly more quantitative and offer higher resolution, but these methods are labor intensive and are unable to offer sequence-level insights as to the exact V(D)J recombination patterns in patients. Solving this problem will enable the wider application of monitoring immune repertoire in clinical settings.

With the development of massively parallel, single-molecule sequencing techniques, it has now become feasible to assay V(D)J recombination by next-generation sequencing, as a means to exhaustively profile the immune repertoire within human subjects. For example, the Roche 454 sequencers has been used to measure and clinically monitor human lymphocyte clonality [10], which takes advantage of 454’s ability to generate longer sequencing reads that potentially covers V(D)J recombination junction points. Another similar study also used Roche 454 to study human T cell subsets [11]. Note that they separated T cell subpopulations and focused on TCR loci only. However, other investigators have focused on Illumina Genome Analyzer or HiSeq that generates only ~50 bp to ~100 bp reads. For example, a group has developed a short reads assembly strategy to first assemble 50 bp sequences and then sample the CDR3 diversity in human T lymphocytes from peripheral blood [12,13]. The data analysis involved in such strategy is much less straightforward, but the technology is more accessible and cost-effective in large scale studies. Several additional TCR-Seq studies [13-16] have been extensively reviewed recently. Some of the commonalities include: (1) they almost always used T-cell DNA or RNA as the first starting material; (2) most of the studies use multiplex PCR reactions to enrich the V(D)J recombinations for next-generation sequencing; (3) large-scale T-cell repertoire analysis has been limited to interrogation of a single TCR subunit per sequencing run, although functional antigen-engaging TCRs are heterodimeric proteins comprising both an α and a ß chain.

In the current study, we explored the feasibility of using ligation-anchored PCR reaction, rather than multiplex PCR reactions, for unbiased amplification of TCR-beta (TRB) repertoire. The key aspect of our approach is ligating a synthesized linker oligo sequence (/5Phos/NNN…NNN/3ddC/) to the 3’end of single-stranded cDNA, thus it is applicable to extracted RNA samples. Additionally, similar to our previous study examining immunoglobulin repertoire [17], instead of relying on flow cytometry or magnetic beads to isolate T cells or B cell populations from peripheral blood, we attempted to assay RNAs extracted from whole blood directly. Finally, we evaluated sequencing data from the latest Illumina Hi-Seq2500 sequencer, which can generate 151 bp single-end reads (~160 million reads per run), with a significantly shortened turnaround time (48 hours for Rapid Mode) compared to Illumina Hi-Seq2000 or Roche 454 sequencer. Despite the shorter reads than 454 sequencer or Illumina MiSeq sequencer, 151 bp read length is still long enough to map V(D)J recombination sites, given appropriate primer design for PCR amplification.

To evaluate this approach, we did a pilot study on patients suffered from meningiomas, the most common primary brain tumors in the United States [18,19], and compared differences of the detected TRB repertoire in patients with benign (grade I) and malignant (grade III) tumors to healthy individuals. This tumor arises from the membranous layers surrounding the central nervous system (CNS), and is not subject to blood-brain barrier. Several previous studies have reported the presence of both humoral [20,21] and cellular [22,23] immune responses in patients with meningiomas. Indeed, it has been proposed that frequent antibody response against specific antigens in even benign meningioma can serve as diagnostic targets [20]. The immune response to meningiomas could be similar to the reported expansion of tumor infiltrating lymphocytes in response to cancerous cells [24]. Therefore, in addition to testing the technical feasibility, our pilot study has the added value of investigating whether there is increased clonality with increased severity of cancer, and whether different patients have similar or distinct CDR3 sequences in clonal T cells.

## Results

### Total RNA extraction and PCR amplification of TRB variable region

**Figure 1:**
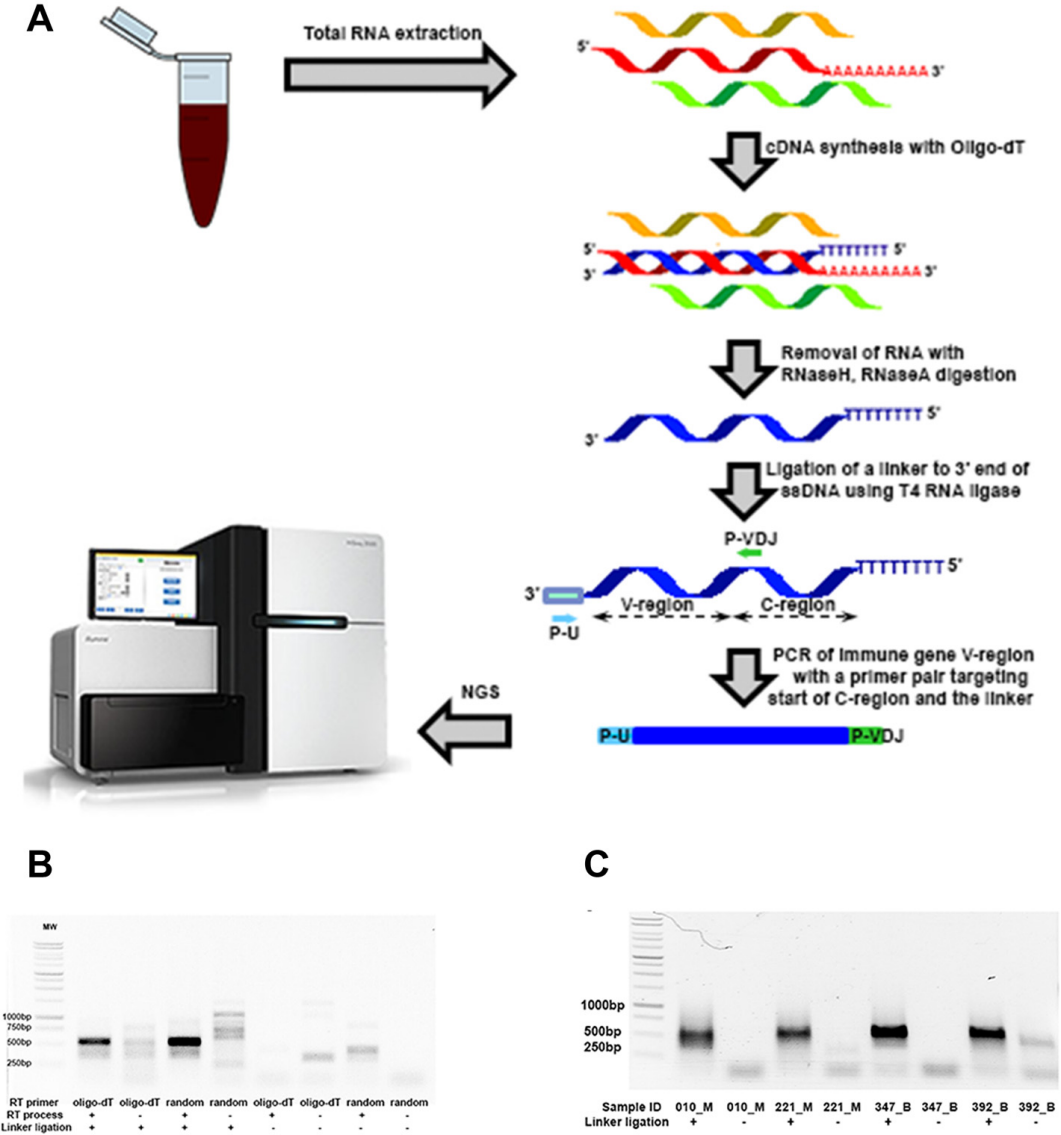
PCR method utilizing single target-specific primer to explore actively transcribed immune genes. **A)**, The scheme of an integrative approach to explore immune repertoire from whole blood (P-U represents a universal primer sitting on the linker, whereas P-VDJ represents a TRB-specific primer targeting the consensus sequence in the constant region right after J-segment); **B)**, DNA gel image of PCR amplified variable region (V-region) of TRB gene from a healthy donor under different conditions; **C)**, DNA gel image of amplicons from malignant and benign patients under linker ligation or control condition.

To explore all the actively transcribed immune receptor variable regions within whole blood in an unbiased manner, we developed an integrative approach to extract total RNA from whole blood, followed by a PCR protocol to capture the variable region (V-region) of specific immune receptor (Figure 1A). In this scheme, after total RNA extraction from whole blood, we generate single-stranded cDNA from reverse transcription of mRNA. After ligation of a synthesized linker oligo sequence to the 3’end of single-stranded cDNA, PCR reaction was carried out using a primer pair (primers P-U and P-VDJ) to target the linker sequence and the consensus starting sequence of immune receptor constant region (C-region), respectively. The P-VDJ primer was specifically designed such that it did not have high sequence homology to any other region other than the TCR locus, and that it avoids any known SNPs. In our proof-of-concept study, total RNA extracted from whole blood of a healthy donor was used to test our methodology on capturing TRB variable region (Figure 1B). For samples that were subject to both reverse transcription (either oligo-dT or random primer mix) and linker ligation, PCR reaction resulted in a predominant band with fragment size around 500 bp, roughly the size expected for TRB variable region. For control samples without reverse transcription, this band was not visible. Additionally, controls without linker ligation did not produce this specific band in DNA gel. Thus, the protocol developed in our study should be able to capture transcribed TRB variants from whole blood directly, without the need to separate T cells first.

### TRB profiling using Illumina high-throughput sequencing

To further evaluate whether the developed protocol is applicable to profile TRB repertoire of clinical samples, we tested the protocol on several blood samples from patients with malignant and benign meningiomas (Additional file 1: Tables 1). The fact that immune responses are present in patients with meningiomas [20,21] suggests that immune receptors such as TCR may respond to this tumor type and serve as a biomarker for diagnosis. We followed the same protocol to profile TRB V-region variants using previously collected whole blood samples (stored at -80°C for more than one year) from two malignant patients (010_M and 221_M) and two benign patients (347_B and 392_B), respectively. Compared to controls without linker ligation, all four reverse transcribed samples with ligation showed the expected predominant band (~500 bp, Figure 1C), suggesting the robustness of this approach. The amplified fragments were further used for next-generation sequencing library preparation. As a control, biological duplicate libraries from two healthy donors (S1 and S2) were also constructed for Illumina sequencing.

Purified PCR amplicons were barcoded with Illumina TruSeq kit for high-throughput sequencing. The latest Illumina HiSeq-2500 sequencer was used for sequencing, with load of ~30% PhiX for proper matrixing and phasing into the lane. Collected sequencing data was filtered, and the reads with either P-U or P-VDJ end tag at the 5’end were selected and mapped using IgBLAST software (see Methods, Additional file 1: Tables 2-7). Reproducibility of the method was measured with Pearson’s correlation for the mapped reads from biological replicate data, and plotted using density scatterplots (Additional file 1: Figure S1). In general, the correlation coefficients were between 0.66 and 0.77 for the biological replicates, suggesting a fair consistency of the data.

### Enrichment of TRB fragments and usage of TRB V-segments

**Figure 2:**
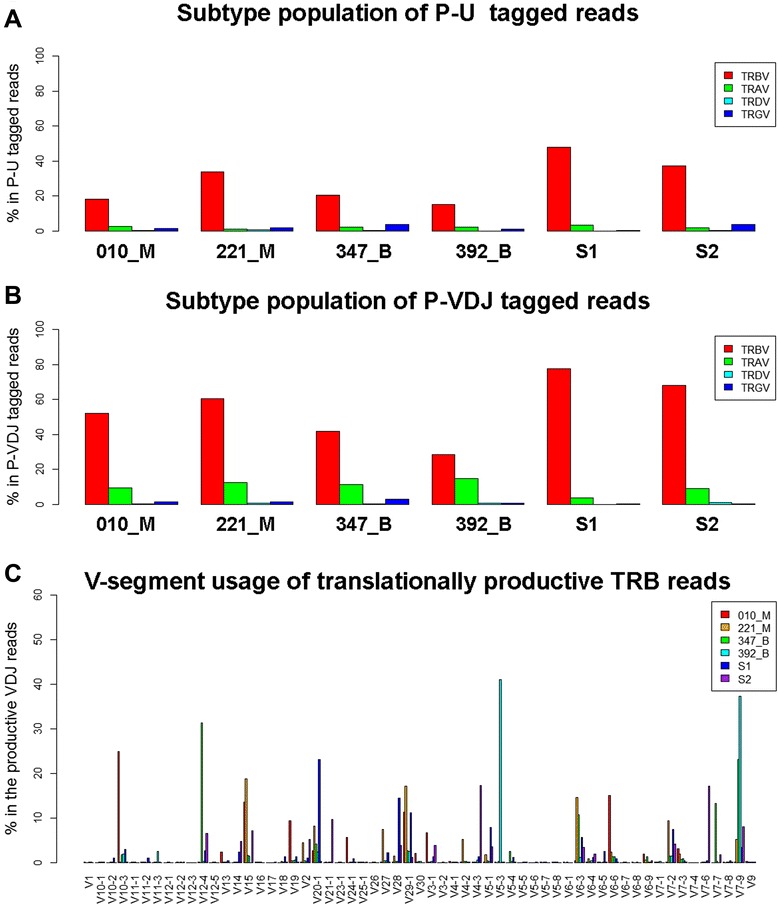
Specificity of the integrative approach on profiling V-region of TRB and V-segment usage. **A &B)**, Subtype population of sequenced reads that are mappable to V-region of TCR (results are based on the reads with PU or P-VDJ end tags); **C)** V-segment usage from V(D)J-containing reads that are translationally productive (no stop codon).

After mapping to germline TCR V gene database using IgBLAST, a significant portion of the reads with either P-U or P-VDJ end tag contained TRB fragments (Figures 2A & 2B). However, we observed a general higher mapping rate for every P-VDJ tagged pool compared to P-U pool from the same dataset. It is possible that higher noise signals from P-U tagged reads were from P-U linker ligation step that the linker sequence can be attached to the 3’end of any template single stranded cDNA molecule. As note, for either P-U or P-VDJ tagged reads, 64% to 96% of the mapped TCRV-containing reads (including TRA, TRB, TRD and TRG) belong to TRB subtype. Therefore, ligation-anchored PCR approach is specific to profile TRB repertoire from blood samples.

We next compared the usage of different V-segments from translationally productive V(D)J reads within P-VDJ tagged read pools (see Methods) from patients with meningiomas and from healthy donors (Figure 2C). We noticed a subset of TRB V-segments with increased usage in meningiomas. V-segments TRBV15, TRBV6-6 and TRBV7-3 showed highest usage in both malignant 010_M and 221_M samples. Specific to individual malignant patient, 010_M showed drastic increase of TRBV10-3, TRBV19, TRBV24-1 and TRBV30, while 221_M had elevated levels of TRBV27 and TRBV4-2 levels. Also noted, TRBV7-9 showed increase of usage in both benign samples, while V12-4, V5-3 and V7-7 levels were greatly increased in either benign individual. Therefore, the statistics of V-segment usage revealed the potential expansion of immune cells with particular TRBV subtypes, possibly reflecting human immune response to meningiomas.

### Exploration of V-J segment pairing in V(D)J recombination

**Figure 3:**
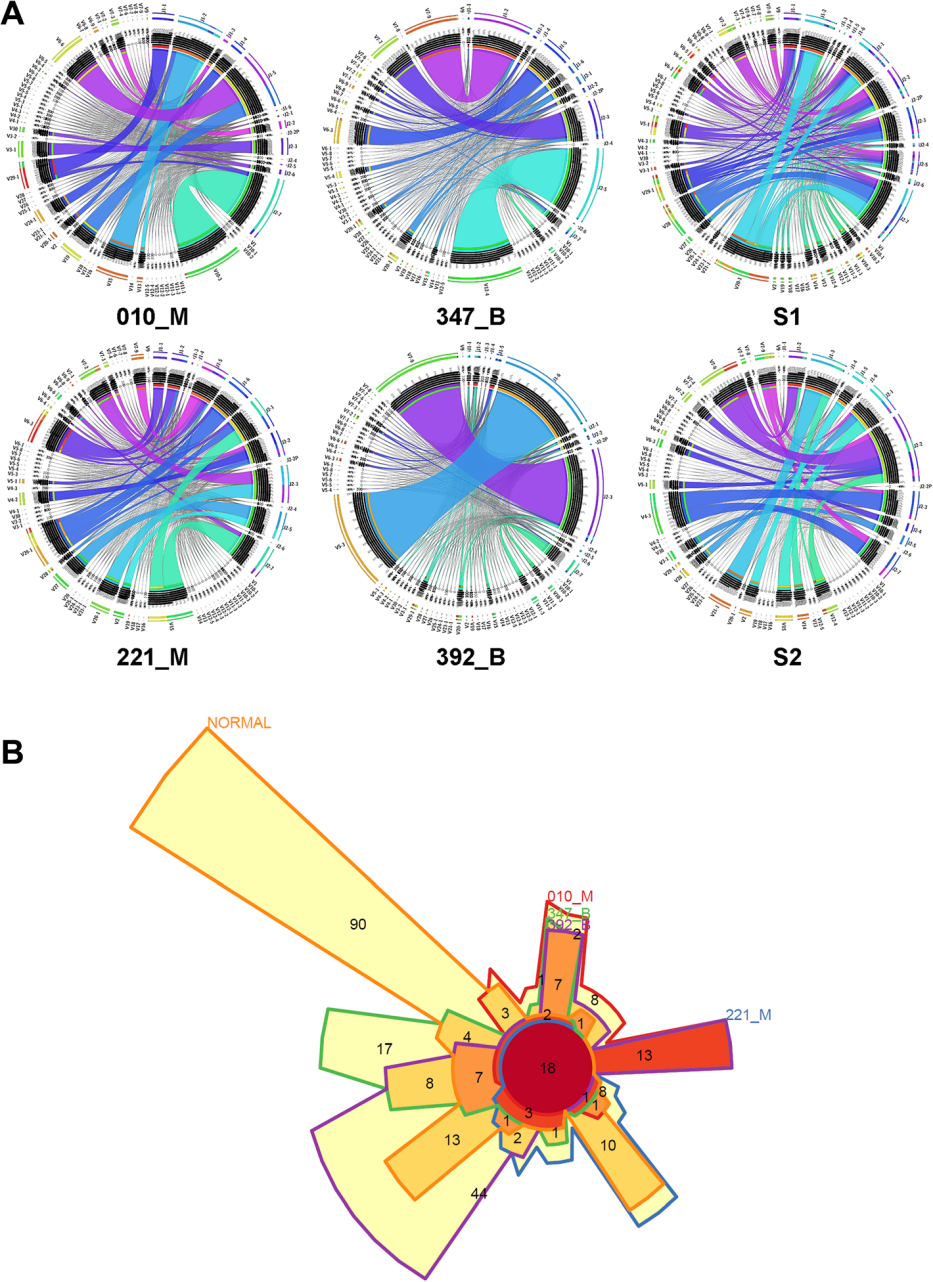
V-J pairing events identified from P-VDJ tagged reads. **A)**, Circos maps illustrating pairing frequencies of V-segments and J-segments from V(D)J-containing reads (all identified segments are ranked by usage frequencies and presented in a clock-wise orientation from the top of the circle, in the order of J-segments and V-segments); **B)**, Chow-Ruskey diagram showing the five-way overlap of V-J pairing events in 010_M, 221_M, 347_B, 392_B with normal pool.

Our method utilized a P-VDJ primer to read through V(D)J junction sequences from the reverse complement strand of the 5’-end common sequence of TRBC1 and TRBC2. With 151 bp read length, the reads can extend to FR3 region of V-segment for most of the V(D)J recombination events. To explore V-J pairing in V(D)J recombination, we counted and normalized pairing frequencies between different V- and J-segments, and presented the results in Circos plots [25] (Figure 3A). Interesting to note, pairing of TRBV7-3 with TRBJ2.2 occurred more frequently in both malignant meningiomas samples (3.02% and 1.83% of total population, respectively) compared to benign ones (0.71% and 0.67%, respectively), and almost abolished in normal controls (less than 0.002%). In contrast, pairing of TRBV5-1 or TRBV7-2 with TRBJ2.2 was enriched in normal controls but not in patient samples. However, in general, the V-J pairing patterns shown with Circos plots appear to be distinct for each patient. The 5-way overlap of V-J pairing events of individual patient with normal control pool was presented in Chow-Ruskey diagram (only events occupying more than 0.01% of total population counted). As shown in Figure 3B, the events detected in normal pool is largely different from those in the patients, with less than half of the V-J pairing overlapped with any patient sample. In addition, a number of events detected in individual patient sample are unique V-J pairing. Also noted, V-J pairing detected in individual malignant patient was less than the pairing in benign or normal samples. In summary, from V-J pairing analysis, it is reasonable to postulate that particular subtype(s) of V(D)J recombinant T cells expand dramatically in patient blood, thus relative weight of other subtype T-cells in the total population decreases accordingly.

### Characterization of “immune signature” – CDR3

In immune system, specific T cell clone(s) can expand dramatically in response to tumor antigens. Counting analysis of V-J pairing (frequencies detected at greater than 0.01% of total population) showed less number of pairing in malignant meningiomas (57 for 010_M, 58 for 221_M) compared to benign meningiomas (82 for 347_B, 121 for 392B) or normal controls (127 for S1, 87 for S2). But when we counted V-J pairing at a cutoff of 5% of total population, we found more pairing events in malignant (7 and 9 events) than benign (4 and 2 events) or normal (3 and 5 events).We then asked whether significantly expanded V-J pairing events in malignant patients could result from clonal expansion of particular T cells in which the sequence of CDR3 region can serve as a signature. We chose the V-J pairing events that are greatly expanded in either malignant individual (greater than 5% of total population) compared to normal controls (less than 0.01% of total population in both normal samples) for CDR3 sequence analysis. In total, eight pairing events TRBV6-6 with TRBJ1-5, TRBV15 with TRBJ1-2, TRBV3-1 with TRBJ2-3, TRBV24-1 with TRBJ1-4, TRBV15 with TRBJ2-7, TRBV29-1 with TRBJ1-6, TRBV7-9 with TRBJ1-5, and TRBV4-2 with TRBJ2-2 were identified as dramatically increased in either one of the malignant samples. Pairing of TRBV7-3 and TRBJ2.2 that showed moderate increase in both malignant samples (greater than 1% of total population) was also included for CDR3 analysis.

**Figure 4:**
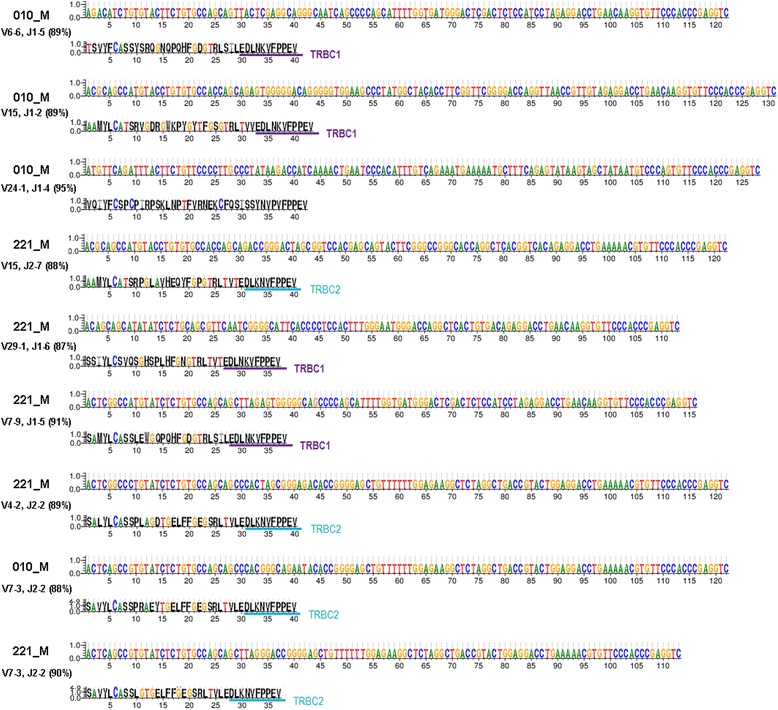
Sequence logos for detected FR3- TRBC portions of malignant meningiomas. Visualized in the DNA sequence logos are the dominant clonal CDR3 sequences of selected V-J pairings (the percentage of dominant clonal reads in the total are also included); the translated protein sequence logos illustrate antigen recognition regions from the end of FR3 and the beginning of TRBC. TRBC1 and TRBC2 sequences are underlined in purple and cyan colors, respectively.

Except for pairing of TRBV3-1 with TRBJ2-3 which has unusual CDR3 skipping, sequencing reads for every other combination showed one dominant FR3-CDR3 junction DNA sequence (Figure 4), which accounts for 87% to 95% of the corresponding pairing event, suggesting clonal expansion. We also generated protein sequence logos for further comparison. In protein sequence logo plots, the amino acid sequences between the conserved Cys (C) at the 3’end of FR3 region (positioned at 6) and the common VFPPEY motif at the 5’end of TRB constant region varied a lot for different V-J pairing events. In malignant meningiomas, both TRBC1 and TRBC2 subtypes were used in those expanded pairing events. As expected, most of the J subtypes (J1 or J2) fused to the closet known TRBC subtypes (TRBC1 or TRBC2). However, the dominant clone for TRBV24-1 with TRBJ1-4 produced an unusual recombination/mutation at the beginning part of TRB constant region that is not mappable to either TRBC1 or TRBC2. For pairing of TRBV7-3 with TRBJ2-2 that showed expansion in both malignant samples, we observed that 88% to 90% of this population was from single dominant CDR3 signature. However, their translated CDR3 sequences were not identical (PRAEY *vs.* LG), suggesting occurrence of other genomic editing events, such as hypermutation. In summary, CDR3 sequence logo analysis identified CDR3 signature sequences associated with individual malignant patient, which may reflect expansion of several specific V-J pairing clones in patient blood.

## Discussion and conclusions

In the current study, we presented an integrated approach by using single primer PCR together with next-generation sequencing to interrogate immune repertoire of TCR-beta. We have demonstrated the technical feasibility to use this system to infer immune repertoire, using whole blood from four meningiomas patients and two healthy donors. By aligning reads to a sequence database of germline V-genes, D-genes and J-genes, the usage of different V-gene segments was quantified. Interestingly, comparison between malignant, benign and normal groups identified an increased usage of TRBV15, TRBV6-6 and TRBV7-3 in malignant meningiomas. However, the pairing of V-J subtypes for recombination revealed a generally diversified immune repertoire for individual patient, although TRBV7-3 with TRBJ2.2 appears to be associated with malignant transformation. Further analysis of CDR3 region sequence logos of the top expanded V-J pairing in malignant meningiomas indicated distinct CDR3 signatures for the two malignant patients. However, we caution that these observations were made on a small number of samples, and they may not have any biological significance. Our purpose is to use these data to demonstrate the technical feasibility of “single-primer” interrogation of immune repertoire, rather than determining what differs between malignant and benign tumors.

There are several unique aspects of our protocol, compared to previous studies. First of all, total RNA is extracted directly from frozen blood samples for profiling, thus the procedure can be easily adapted for clinical application. Second, by using ligation-anchored PCR for amplification, all the recombination events at a particular immune gene locus is likely to be amplified in an unbiased manner. Furthermore, sequencing of barcoded libraries through Illumina Hi-Seq 2500 ensures fast turn-around time (less than 48 hours) and good sequencing depth (~160 million reads per lane) at a relatively low cost. Finally, we recognize that more recent generations of Illumina sequencers can now sequence 250 bp or even continuous 500 bp(2 × 250 bp) reads, potentially further reduce the computational complexity and increase the rate of recovering full length V(D)J recombination for our approach.

There are several major limitations of our protocol as well. First, due to the need to add in 3’-adaptors to the cDNA terminus for ligation-anchored PCR, our method relies on RNA samples, which are less readily available and more vulnerable to degradation, compared to genomic DNA samples. However, in the current study, we used frozen whole blood samples and still obtained satisfactory results, suggesting that it is practically feasible to use this method in real-world clinical settings. Second, although Illumina Hi-Seq 2500 provided longer read length (151 bp) than Illumina Hi-Seq 2000 (101 bp) to cover immune signature region CDR3, longer read length is still needed to cover the entire variable region of immune genes. Thus the latest Illumina MiSeq with 250 bp or 2x250 bp chemistry should be more suitable for profiling immune repertoire. Third, our sequencing data showed higher sequencing depth of malignant sample libraries (010_M, 221_M) compared to benign ones (Additional file 1: Table 2). We acknowledge that uneven mixing of the constructed libraries for sequencing could be the reason. To address the issue of sequencing depth, we performed additional data analysis using the first 7 million sequencing reads of 010_M and 221_M data (Additional file 1: Table 4&5). Of note, the results from the 7-million-read data were consistent with the conclusions from the whole sequencing data, indicating that the sequencing depth in our study is deep enough. Fourth, we specifically designed primers to target TCR-beta isoforms. Our sequencing data contained some other TCR isoforms as well, suggesting the presence of some minor cross reactions. Our future studies may design specific primers for other types of isoforms as well, to investigate whether it is feasible to interrogate all major TCR isoforms in the same sequencing run. Finally, the sample size in our study is small, which prevents us to draw any statistically significant conclusions on clonal expansion of TRB subtypes associated with malignant transformation of meningiomas; however, the results from high-throughput sequencing data analysis do support validity of the developed integrated approach.

In conclusion, we have developed a ligation-anchored PCR approach to interrogate immune repertoire, and demonstrated its technical feasibility and effectiveness in capturing the TCR-beta landscapes. Recently, human antibody repertoire has been reported to change in response to influenza vaccination [26]. We expect further development of our technology may enable a comprehensive delineation of immune repertoire, including other forms of TCRs as well as immunoglobulins.

## Methods

### Sample collection, RNA extraction and cDNA synthesis

Peripheral blood from patients with meningiomas from the USC Brain Tumor Bank was collected for the study. The study was approved by the Institutional Review Board at the University of Southern California. Informed written consent was obtained from the study subjects to participate in the study and to publish their sequence data. Demographic information was obtained from patient medical records. For assay consistency evaluation, peripheral blood from two healthy donors was also collected. Standard “purple-top” EDTA tubes (BD Vacutainer) were used for blood extraction and storage. The Institutional Review Board (IRB) at the University of Southern California reviewed and approved the study. Informed consent was obtained for all participants.

Total RNA was extracted from ice thawed 400 μL of frozen blood (stored at -80°C) using TRIzol extraction reagent (Life Technologies, Grand Island, NY). For each TRIzol extracted RNA sample, 2mL of anhydrous ethanol was added before passing through an RNeasy-mini column (QIAGEN, Valencia, CA) with in-column DNase I digestion performed. Purified total RNA was eluted in ddH_2_O for further cDNA synthesis using M-MuLV reverse transcriptase kit (NEB, Ipswich, MA) following the recommended protocol, and stored at -20°C.

### Linker ligation and PCR amplification of TRB variants

For each reverse transcribed product, AMPure XP magnetic beads (Beckman Coulter, Danvers, MA) was added to the mixture, followed by 80% ethanol wash to remove primers introduced in reverse transcription step. Each sample was eluted in 30 μL EB buffer (QIAGEN, Valencia, CA) with 1 μL RNase H (10U /μL, Epicentre, Madison, WI) and 2 μL RNase A (5 μg/μL, Epicentre, Madison, WI) added to digest RNA templates for 1 hour at 37C, followed by a heat-inactivation step for 5 min. at 95°C.

Ligation of the linker primer to RNase-treated cDNA sample (ssDNA) was carried out in a total volume of 60 μL using T4 RNA ligation kit (Promega, Madison, WI) following vendor recommended protocol. The linker primer was synthesized (Integrated DNA Technologies, San Diego, CA) based on the sequence and modification in a previous study [27]. The mixture was incubated overnight at room temperature. For control reaction, ddH_2_O instead of T4 RNA ligase (Promega, Madison, WI) was added.

After overnight incubation, the ligation product was incubated with AMPure XP beads for magnetic purification. Each sample was eluted in 20 μL ddH_2_O. PCR reaction was performed using HotStart PCR reagent kit (EMD Chemicals, Billerica, MA) with 2 μL of ligated ssDNA as PCR template in 50 μL reaction (MgSO_4_ 1.5mM final concentration, dNTPs 0.2 mM each final, Hot Start DNA Polymerase 2 μL). In a reaction mixture without primer loaded, a universal primer targeting the linker (GCG GCC GCT TAT TAA CCC, final concentration 1 μM) was first added for 2^nd^ strand synthesis at 95°C for 2.5 min., 70°C for 5min. extension, and 4°C forever. Then another primer targeting the consensus sequence of TRB constant region (GAC CTC GGG TGG GAA CAC, final concentration 1 μM) was added, and the PCR tubes were incubated at 95°C for 2min., followed by 40 cycles of PCR reactions at 95°C for 20 sec., 65°C for 10 sec. and 70°C for 30 sec. before cooling down to 4°C. All PCR reactions were done in a gradient PCR machine (Eppendorf Mastercycler).

### NGS library preparation and sequencing

Illumina TruSeq Sample Prep Kit (Illumina, San Diego, CA) was used for library preparation. Briefly, amplicons were purified before sequential steps of end-repair, adenylation of 3’end and adaptor ligation using the recommended protocol. Finally, the barcoded libraries were enriched with 10 cycles of PCR reactions and cleaned with AMPure XP beads. The quality of prepared libraries was assessed using Agilent High Sensitivity DNA Chip on a Bioanalyzer (Agilent Technologies, Santa Clara, CA). The bar-coded libraries were quantified with KAPA Library Quantification Kit (Kapa Biosystems, Woburn, MA) before mixing for sequencing in a single lane on the Illumina HiSeq 2500 system. The raw FASTQ data files of 151 bp single-end reads were collected for downstream analysis.

### Analysis of sequencing data

For the raw FASTQ data collected from high-throughput sequencing, sequencing quality analysis was first performed using FASTQC (http://www.bioinformatics.babraham.ac.uk/projects/fastqc/) to ensure the mean values of sequence quality (Phred Score) for each base is greater than 32. Then a custom PERL script was used to select the reads with the starting sequences containing either P-U (with additional 5 bp extended to the ligation site) or P-VDJ primer sequence. Unlike genome or exome sequencing, the nature of sequencing data from immune gene variable regions requires 1) mapping against a sequence database containing V-gene, D-gene and J-gene segments; and 2) aligning the V(D)J junctions formed. For mapping of the sequenced reads, recently released IgBLAST [28] was employed to identify reads that can be mapped to germline V, V-D, V-J, or V-D-J segments. The default parameters were used for mapping, and only the V, D and J subtypes with highest similarity were outputted. Due to lack of a standalone version of IgBLAST for TCR gene analysis, the sequencing data (reformatted to FASTA files) were split into small batches and uploaded to the IgBLAST web server for mapping. Biological duplicate data of two healthy donors were mapped separately, and the reproducibility between replicates was analyzed (Additional file 1: Figure S1). The mapping results from the duplicate data were further concatenated for all other analysis.

Sequence logo graphs were generated using standalone version of WebLogo (weblogo.berkeley.edu) [29]. Circos maps were plotted using Circos Table Viewer module in Circos Tools software (http://circos.ca/software/download/) [25]. Briefly, Circos Tools package was downloaded to a Linux workstation and installed. Text-style VJ recombination matrix files were processed with *parse-table* and *make-conf* commands to generate Circos input files. Then *circos* command was used to generate the plots with all default parameters except *label_font* changed to “condensed”, *label_parallel* changed to “no”, *label_size* changed to 40, *segment_order* changed to “ascii”, *use_segment_normalization* changed to “yes”, and *segment_normalization_function* changed to “total”. All other plots were generated using R.

## Additional file

Additional file 1: Figure S1. Correlations of TRBV subtype usage and VJ recombination usage identified in duplicate libraries of normal samples. **Table 1.** Demographics of Patients with Meningiomas **Table 2.** Summary of sequencing data statistics for meningiomas samples **Table 3.** Summary of V(D)J junction reads for meningiomas samples **Table 4.** Summary of statistics of the 7-million-read sequencing data for meningiomas samples **Table 5.** Summary of V(D)J junction reads of the 7-million-read sequencing data for meningiomas samples **Table 6.** Summary of sequencing data statistics for normal samples **Table 7.** Summary of V(D)J junction reads for normal samples.
