# Supplementary material for: Ligation-anchored PCR unveils immune repertoire of TCR-beta from whole blood
Source: BMC Biotechnol. 2015 May 28;15:39. doi: 10.1186/s12896-015-0153-9 (PMC4446965; doi:10.1186/s12896-015-0153-9)
Supplement: Additional file 1: Figure S1. — Correlations of TRBV subtype usage and VJ recombination usage identified in duplicate libraries of normal samples. Table 1. Demographics of Patients with Meningiomas Table 2. Summary of sequencing data statistics for meningiomas samples Table 3. Summary of V(D)J junction reads for meningiomas samples Table 4. Summary of statistics of the 7-million-read sequencing data for meningiomas samples Table 5. Summary of V(D)J junction reads of the 7-million-read sequencing data for meningiomas samples Table 6. Summary of sequencing data statistics for normal samples Table 7. Summary of V(D)J junction reads for normal samples. [file 12896_2015_153_MOESM1_ESM.doc]

# Ligation-anchored PCR unveils immune repertoire of TCR-beta from whole blood

Fan Gao^1#^ & Kai Wang ^1, 2*^

^1^: Zilkha Neurogenetic Institute, University of Southern California, Los Angeles, CA 90089, USA

^2^: Department of Psychiatry, University of Southern California, Los Angeles, CA 90089, USA

^#^: Current address: The Picower Institute for Learning and Memory, MIT, Boston, MA 02139, USA

*: Correspondence should be addressed to Dr. Kai Wang, 1501 San Pablo Street, Los Angeles, CA 90089, USA, [kaiwang@usc.edu](mailto:kaiwang@usc.edu)

Emails: Fan Gao [fangao@mit.edu](mailto:fangao@mit.edu), Kai Wang [kaiwang@usc.edu](mailto:kaiwang@usc.edu)

**Supplementary information**

**Supplementary Figure: Density scatterplots of TRBV subtype usage and VJ recombination usage identified in the biological duplicates of normal samples S1 & S2. Pearson’s correlation coefficients were also shown in the plots. Log_10_(counts+1) values were used for calculation and plotting.**


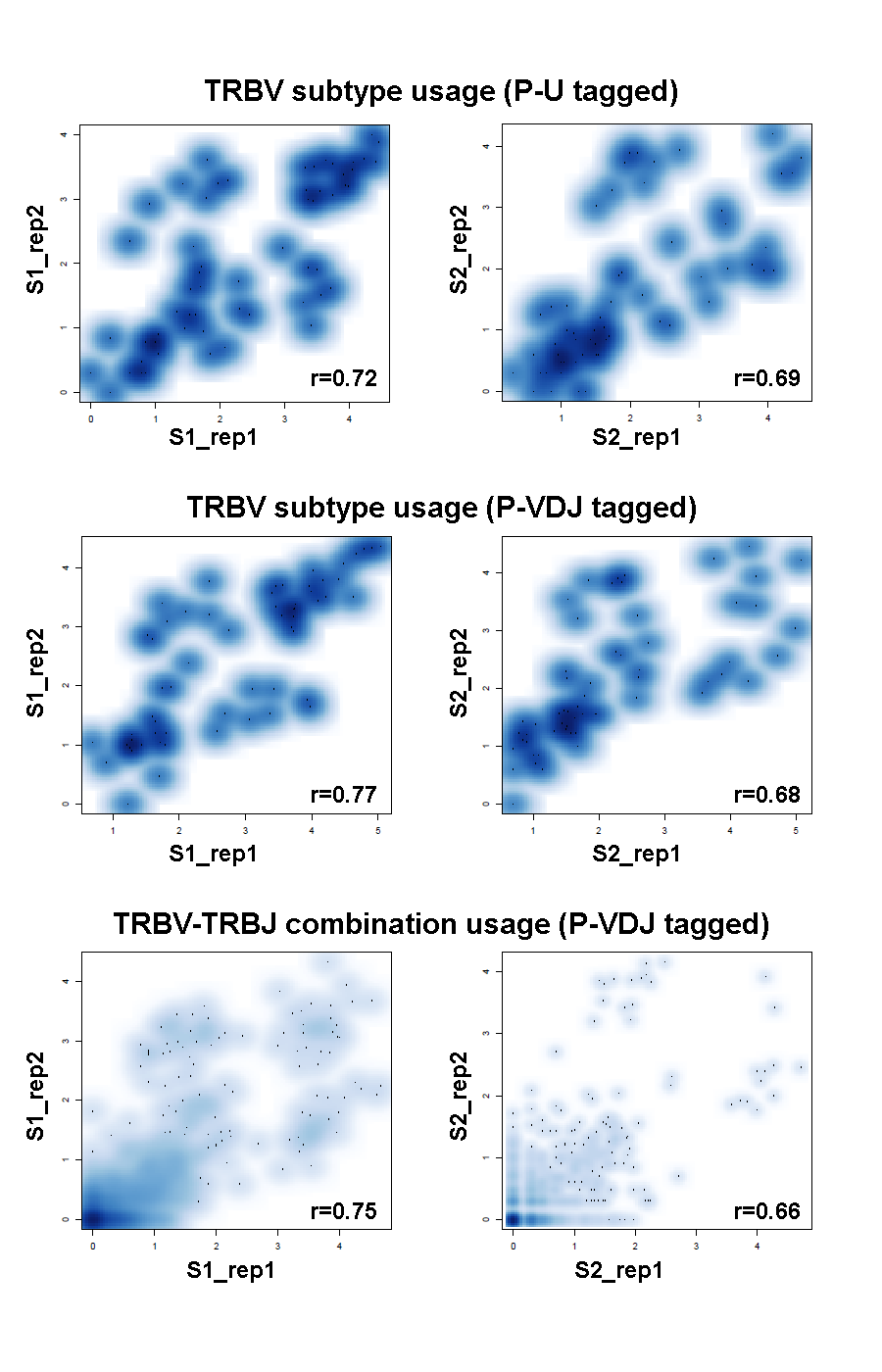


**Supplementary Table 1. Demographics of Patients with Meningiomas**

| Sample ID | Gender | Race | Chemo/ Radiation | WHO Grade | Sample Type |
| --- | --- | --- | --- | --- | --- |
| 347_B | F | H | NONE | I | Benign |
| 392_B | F | H | NONE | I | Benign |
| 010_M | M | C | NONE | III | Malignant |
| 221_M | M | H | NONE | III | Malignant |

Supplementary Table 2. Summary of sequencing data statistics for meningiomas samples

| **Sample ID** | **010_M** | **221_M** | **347_B** | **392_B** |
| --- | --- | --- | --- | --- |
| Total number of sequenced reads | 36,794,407 | 13,405,950 | 5,807,826 | 9,051,764 |
| Number of reads with P-U end tag | 14,714,739 | 5,540,304 | 1,493,598 | 2,267,131 |
| Number of reads with P-VDJ end tag | 9,933,135 | 5,177,620 | 1,211,356 | 2,777,050 |
| P-U tagged reads containing TRBV (+)^*^ | 2,663,062 | 1,880,539 | 304,399 | 338,750 |
| P-U tagged reads containing TRAV (+) | 347,758 | 44,272 | 33,608 | 44,525 |
| P-U tagged reads containing TRDV (+) | 9,510 | 28,161 | 939 | 689 |
| P-U tagged reads containing TRGV (+) | 208,117 | 105,975 | 56,620 | 18,033 |
| P-VDJ tagged reads containing TRBV (-)^*^ | 5,164,687 | 3,137,902 | 505,981 | 787,828 |
| P-VDJ tagged reads containing TRAV (-) | 916,075 | 638,292 | 135,112 | 410,918 |
| P-VDJ tagged reads containing TRDV (-) | 6,799 | 26,969 | 867 | 13,765 |
| P-VDJ tagged reads containing TRGV (-) | 145,852 | 61,022 | 37,225 | 12,931 |

*(+) Reads aligned to the coding strand; (-) Reads aligned to the reverse complement strand.

**Supplementary Table 3. Summary of V(D)J junction reads for meningiomas samples**

| Sample ID | 010_M | 221_M | 347_B | 392_B |
| --- | --- | --- | --- | --- |
| P-VDJ tagged reads containing productive TRB-V(D)J junctions (-) | 1,177,610 | 1,250,012 | 129,594 | 127,592 |
| P-VDJ tagged reads containing productive TRA-V(D)J junctions (-) | 185,624 | 9,341 | 4,047 | 14,328 |
| P-VDJ tagged reads containing productive TRD-V(D)J junctions (-) | 1,635 | 3,466 | 88 | 186 |
| P-VDJ tagged reads containing productive TRG-V(D)J junctions (-) | 2,750 | 166 | 192 | 260 |

# Supplementary Table 4. Summary of statistics of the 7-million-read sequencing data for meningiomas samples

| Sample ID | 010_M (7M reads) | 221_M (7M reads) | 347_B | 392_B |
| --- | --- | --- | --- | --- |
| Total number of sequenced reads | 7,000,000 | 7,000,000 | 5,807,826 | 9,051,764 |
| Number of reads with P-U end tag | 2,832,252 | 2,928,413 | 1,493,598 | 2,267,131 |
| Number of reads with P-VDJ end tag | 1,889,027 | 2,712,692 | 1,211,356 | 2,777,050 |
| P-U tagged reads containing TRBV (+)^*^ | 512,879 | 994,352 | 304,399 | 338,750 |
| P-U tagged reads containing TRAV (+) | 67,062 | 22,906 | 33,608 | 44,525 |
| P-U tagged reads containing TRDV (+) | 1,825 | 14,776 | 939 | 689 |
| P-U tagged reads containing TRGV (+) | 39,981 | 55,806 | 56,620 | 18,033 |
| P-VDJ tagged reads containing TRBV (-)^*^ | 979,048 | 1,646,272 | 505,981 | 787,828 |
| P-VDJ tagged reads containing TRAV (-) | 174,039 | 336,225 | 135,112 | 410,918 |
| P-VDJ tagged reads containing TRDV (-) | 1,251 | 14,223 | 867 | 13,765 |
| P-VDJ tagged reads containing TRGV (-) | 27,817 | 32,075 | 37,225 | 12,931 |

*(+) Reads aligned to the coding strand; (-) Reads aligned to the reverse complement strand.

**Supplementary Table 5. Summary of V(D)J junction reads of the 7-million-read sequencing data for meningiomas samples**

| Sample ID | 010_M (7M reads) | 221_M (7M reads) | 347_B | 392_B |
| --- | --- | --- | --- | --- |
| P-VDJ tagged reads containing productive TRB-V(D)J junctions (-) | 224,383 | 651,607 | 129,594 | 127,592 |
| P-VDJ tagged reads containing productive TRA-V(D)J junctions (-) | 34,890 | 4,253 | 4,047 | 14,328 |
| P-VDJ tagged reads containing productive TRD-V(D)J junctions (-) | 294 | 1,799 | 88 | 186 |
| P-VDJ tagged reads containing productive TRG-V(D)J junctions (-) | 486 | 93 | 192 | 260 |

Supplementary Table 6. Summary of sequencing data statistics for normal samples

| **Sample ID** | **S1_rep1** | **S1_rep2** | **S2_rep1** | **S2_rep2** |
| --- | --- | --- | --- | --- |
| Total number of sequenced reads | 1,741,581 | 541,838 | 1,598,845 | 570,634 |
| Number of reads with P-U end tag | 402,566 | 150,445 | 400,529 | 140,370 |
| Number of reads with P-VDJ end tag | 670,897 | 254,445 | 592,434 | 194,034 |
| P-U tagged reads containing TRBV (+)^*^ | 193,397 | 70,541 | 128,042 | 73,034 |
| P-U tagged reads containing TRAV (+)^*^ | 16,444 | 1,154 | 9,584 | 549 |
| P-U tagged reads containing TRDV (+)^*^ | 11 | 7 | 631 | 19 |
| P-U tagged reads containing TRGV (+)^*^ | 1,320 | 395 | 18,020 | 1,121 |
| P-VDJ tagged reads containing TRBV (-)^*^ | 541,060 | 177,998 | 406,401 | 127,632 |
| P-VDJ tagged reads containing TRAV (-)^*^ | 24,673 | 8,316 | 33,761 | 36,295 |
| P-VDJ tagged reads containing TRDV (-)^*^ | 121 | 80 | 5,831 | 503 |
| P-VDJ tagged reads containing TRGV (-)^*^ | 315 | 94 | 211 | 606 |

*(+) Reads aligned to the coding strand; (-) Reads aligned to the reverse complement strand.

**Supplementary Table 7. Summary of V(D)J junction reads for normal samples**

| **Sample ID** | **S1_rep1** | **S1_rep2** | **S2_rep1** | **S2_rep2** |
| --- | --- | --- | --- | --- |
| P-VDJ tagged reads containing productive TRB-V(D)J junctions (-) | 416,296 | 138,374 | 113,830 | 80,757 |
| P-VDJ tagged reads containing productive TRA-V(D)J junctions (-) | 12,330 | 2,346 | 14,530 | 3,492 |
| P-VDJ tagged reads containing productive TRD-V(D)J junctions (-) | 40 | 20 | 35 | 181 |
| P-VDJ tagged reads containing productive TRG-V(D)J junctions (-) | 36 | 10 | 12 | 28 |
